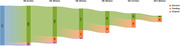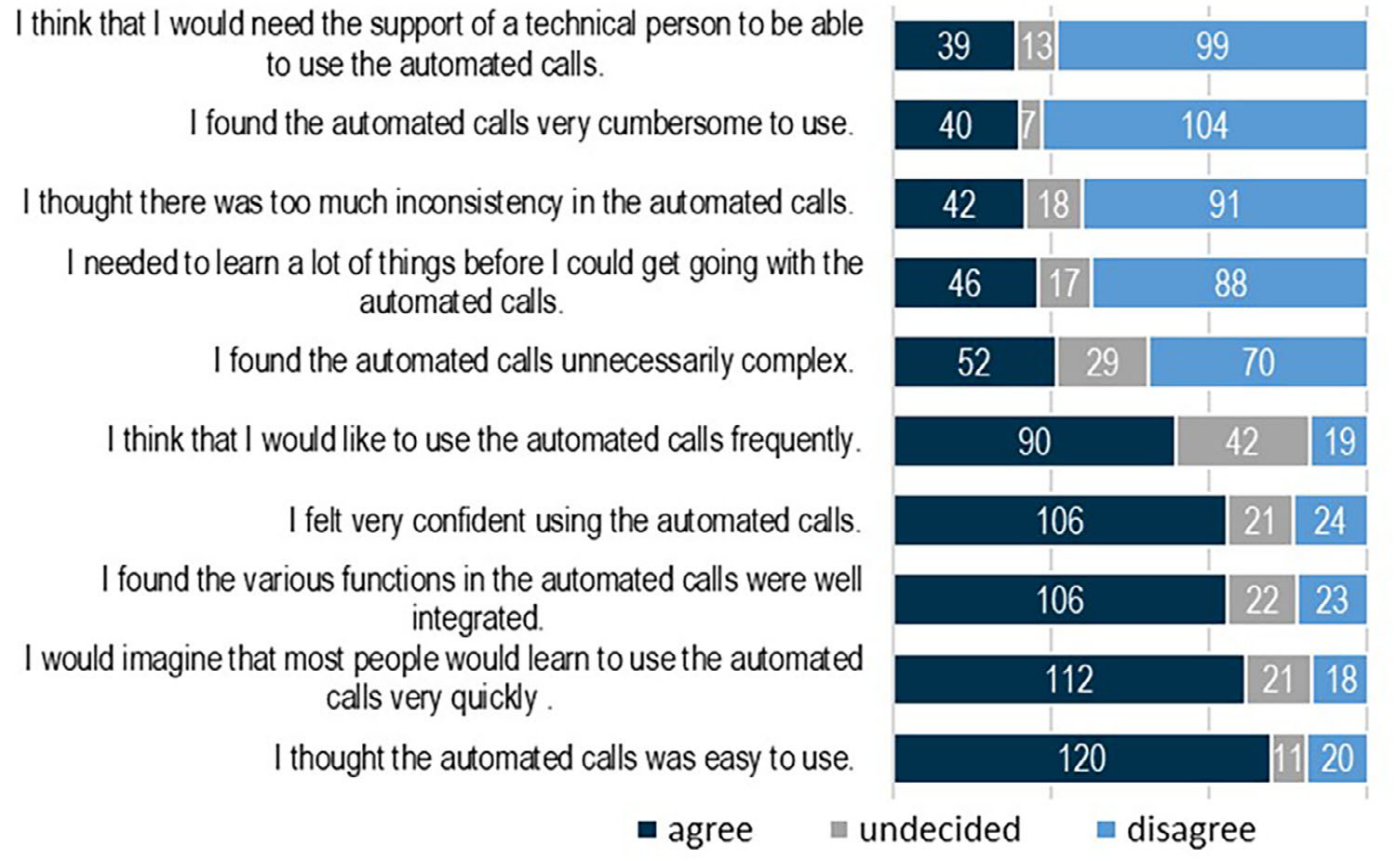# Digital biomarker co‐design with patients and caregivers

**DOI:** 10.1002/alz.084095

**Published:** 2025-01-09

**Authors:** Stefanie Köhler, Alexandra König, Nicklas Linz, Slawek Altenstein, Michaela Butryn, Wenzel Glanz, Frank Jessen, Matthias H. J. Munk, Antje Osterrath, Björn H. Schott, Annika Spottke, Stefan Teipel

**Affiliations:** ^1^ Deutsches Zentrum für Neurodegenerative Erkrankungen e. V. (DZNE) Rostock/Greifswald, Rostock Germany; ^2^ ki:elements GmbH, Saarbrücken Germany; ^3^ CoBTek (Cognition‐Behaviour‐Technology), Université Côte d'Azur, Nice France; ^4^ Department of Psychiatry and Psychotherapy, Charité, Berlin Germany; ^5^ Medical Faculty University Hospital Magdeburg, Magdeburg Germany; ^6^ University Hospital Cologne, Cologne Germany; ^7^ Deutsches Zentrum für Neurodegenerative Erkrankungen e. V. (DZNE) Bonn, Bonn Germany; ^8^ Excellence Cluster on Cellular Stress Responses in Aging‐Associated Diseases (CECAD), University of Cologne, Cologne Germany; ^9^ Department of Psychiatry and Psychotherapy, University of Tuebingen, Tuebingen Germany; ^10^ Systems Neurophysiology, Department of Biology, Darmstadt University of Technology, Darmstadt Germany; ^11^ German Center for Neurodegenerative Diseases (DZNE), Tuebingen Germany; ^12^ Deutsches Zentrum für Neurodegenerative Erkrankungen e.V., Dresden Germany; ^13^ University Hospital Carl‐Gustav‐Carus Dresden, Dresden Germany; ^14^ Department of Psychiatry and Psychotherapy, University Medical Center Goettingen (UMG), Göttingen Germany; ^15^ Deutsches Zentrum für Neurodegenerative Erkrankungen e.V., Göttingen Germany; ^16^ University of Bonn Medical Center, Bonn Germany; ^17^ University Medical Center Rostock, Rostock Germany

## Abstract

**Background:**

Using artificial intelligence approaches enable automated assessment and analysis of speech biomarkers for Alzheimer’s disease, for example using chatbot technology. However, current chatbots often are unsuitable for people with cognitive impairment. Here, we implemented a user‐centred‐design approach to evaluate and improve usability of a chatbot system for automated speech assessments for people with preclinical, prodromal and early dementia.

**Method:**

Participants (n = 221 by January 2024, 300 planned, see Figure 1) of the PROSPECT‐AD study were automatically called for six times every three months by our chatbot “Mili”. In each call three cognitive tasks (Wordlist, Semantic Verbal Fluency, Story Telling) are offered. We applied the System Usability Scale (SUS) in all participants and conducted additional semi‐structured interviews in a subgroup to gain in‐depth information for improving Mili and adapting it to the users’ needs. During the interviews, we focus on affinity for technology and Mili’s usability.

**Result:**

We collected usability data at M3 by the SUS from 163 participants (12 missings, ØAge: 73.2 years, SD: 6.6 years, see Figure 2) after their second call. The results indicated a good usability of Mili (ØSUS: 70.4/100, SD: 22.2). Within semi‐structured interviews we asked 22 participants (ØAge: 73.0 years, 12 female, ØMMST: 29.3, range 24‐30, medium tech‐savvy) for their ideas to improve the Mili system. We analysed the interviews using qualitative content analysis. Participants wished for an intelligent chatbot and a feedback system after each call about participants’ cognitive performance. In the event that the performance would indicate cognitive decline, Mili should provide information on local services for dementia, and give recommendations for prevention and advises for the user’s physician for medication. Seventeen interviewees (2 undecided, 3 negative) would like to use Mili regularly if they would receive a feedback on their cognitive performance.

**Conclusion:**

Our results revealed a high usability and feasibility of the automated phone calls by Mili. Eligible feedback solutions would increase the usefulness and usage of remote testing if a they provide individual advises for prevention and contact persons. Unrealistic expectations on Mili regarding the recommendation of medication must be discussed with the users.